# A Comprehensive Framework to Evaluate Websites: Literature Review and Development of GoodWeb

**DOI:** 10.2196/14372

**Published:** 2019-10-24

**Authors:** Rosalie Allison, Catherine Hayes, Cliodna A M McNulty, Vicki Young

**Affiliations:** 1 Public Health England Gloucester United Kingdom

**Keywords:** user experience, usability, human-computer interaction, software testing, quality testing, scoping study

## Abstract

**Background:**

Attention is turning toward increasing the quality of websites and quality evaluation to attract new users and retain existing users.

**Objective:**

This scoping study aimed to review and define existing worldwide methodologies and techniques to evaluate websites and provide a framework of appropriate website attributes that could be applied to any future website evaluations.

**Methods:**

We systematically searched electronic databases and gray literature for studies of website evaluation. The results were exported to EndNote software, duplicates were removed, and eligible studies were identified. The results have been presented in narrative form.

**Results:**

A total of 69 studies met the inclusion criteria. The extracted data included type of website, aim or purpose of the study, study populations (users and experts), sample size, setting (controlled environment and remotely assessed), website attributes evaluated, process of methodology, and process of analysis. Methods of evaluation varied and included questionnaires, observed website browsing, interviews or focus groups, and Web usage analysis. Evaluations using both users and experts and controlled and remote settings are represented. Website attributes that were examined included usability or ease of use, content, design criteria, functionality, appearance, interactivity, satisfaction, and loyalty. Website evaluation methods should be tailored to the needs of specific websites and individual aims of evaluations. GoodWeb, a website evaluation guide, has been presented with a case scenario.

**Conclusions:**

This scoping study supports the open debate of defining the quality of websites, and there are numerous approaches and models to evaluate it. However, as this study provides a framework of the existing literature of website evaluation, it presents a guide of options for evaluating websites, including which attributes to analyze and options for appropriate methods.

## Introduction

### Background

Since its conception in the early 1990s, there has been an explosion in the use of the internet, with websites taking a central role in diverse ﬁelds such as ﬁnance, education, medicine, industry, and business. Organizations are increasingly attempting to exploit the benefits of the World Wide Web and its features as an interface for internet-enabled businesses, information provision, and promotional activities [1,2]. As the environment becomes more competitive and websites become more sophisticated, attention is turning toward increasing the quality of the website itself and quality evaluation to attract new and retain existing users [3,4]. What determines website quality has not been conclusively established, and there are many different deﬁnitions and meanings of the term quality, mainly in relation to the website’s purpose [5]. Traditionally, website evaluations have focused on usability, deﬁned as “the extent to which a product can be used by speciﬁed users to achieve speciﬁed goals with effectiveness, efficiency and satisfaction in a speciﬁed context of use [6].” The design of websites and users’ needs go beyond pure usability, as increased engagement and pleasure experienced during interactions with websites can be more important predictors of website preference than usability [7-10]. Therefore, in the last decade, website evaluations have shifted their focus to users’ experience, employing various assessment techniques [11], with no universally accepted method or procedure for website evaluation.

### Objectives

This scoping study aimed to review and define existing worldwide methodologies and techniques to evaluate websites and provide a simple framework of appropriate website attributes, which could be applied to future website evaluations.

A scoping study is similar to a systematic review as it collects and reviews content in a field of interest. However, scoping studies cover a broader question and do not rigorously evaluate the quality of the studies included [12]. Scoping studies are commonly used in the fields of public services such as health and education, as they are more rapid to perform and less costly in terms of staff costs [13]. Scoping studies can be precursors to a systematic review or stand-alone studies to examine the range of research around a particular topic.

The following research question is based on the need to gain knowledge and insight from worldwide website evaluation to inform the future study design of website evaluations: what website evaluation methodologies can be robustly used to assess users’ experience?

To show how the framework of attributes and methods can be applied to evaluating a website, e-Bug, an international educational health website, will be used as a case scenario [14].

## Methods

This scoping study followed a 5-stage framework and methodology, as outlined by Arksey and O’Malley [12], involving the following: (1) identifying the research question, as above; (2) identifying relevant studies; (3) study selection; (4) charting the data; and (5) collating, summarizing, and reporting the results.

### Identifying Relevant Studies

Following the Preferred Reporting Items for Systematic Reviews and Meta-Analyses guidelines [15], studies for consideration in the review were located by searching the following electronic databases: Excerpta Medica dataBASE, PsycINFO, Cochrane, Cumulative Index to Nursing and Allied Health Literature, Scopus, ACM digital library, and IEEE Xplore SPORTDiscus. The keywords used referred to the following:

- Population: websites
- Intervention: evaluation methodologies
- Outcome: user’s experience.

Table 1 shows the specific search criteria for each database. These keywords were also used to search gray literature for unpublished or working documents to minimize publication bias.

**Table 1:** Full search strategy used to search each electronic database.

Database	Search criteria	Note	Hits (n)
EMBASE^a^	(web* OR internet OR online) AND (user test* OR heuristic	Search on the field “Title”	689
PsychINFO	evaluation OR usability OR evaluation method* OR measur* OR eye-track* OR eye track*	Search on the field “Title”	816
Cochrane	OR metric* OR rat* OR rank* OR question* OR survey OR stud* OR thinking aloud OR think aloud OR observ* OR complet* OR evaluat*	Search on the fields “Title, keywords, abstract”	1004
CINAHL^b^	OR attribut* OR task*) AND (satisf* OR quality OR efficien* OR task efficiency OR effective* OR appear* OR content	Search on the field “Title”	263
Scopus	OR loyal* OR promot* OR adequa* OR eas* OR user* OR experien*);	Search on the field “Title”	3714
ACM^c^ Digital Library	Publication date=between 2006 and 2016; Language published in=English	Search on the field “Title”	89
IEEE^d^ Xplore	(web) AND (evaluat) AND (satisf* OR user* OR quality*); Publication date=between 2006 and 2016; Language published in=English	Search on the field “Title”	82

^a^EMBASE: Excerpta Medica database.

^b^CINAHL: Cumulative Index to Nursing and Allied Health Literature.

^c^ACM: Association for Computing Machinery.

^d^IEEE: Institute of Electrical and Electronics Engineers.

### Study Selection

Once all sources had been systematically searched, the list of citations was exported to EndNote software to identify eligible studies. By scanning the title, and abstract if necessary, studies that did not fit the inclusion criteria were removed by 2 researchers (RA and CH). As abstracts are not always representative of the full study that follows or capture the full scope [16], if the title and abstract did not provide sufficient information, the full manuscript was examined to ascertain whether they met all the inclusion criteria, which included (1) studies focused on websites, (2) studies of evaluative methods (eg, use of questionnaire and task completion), (3) studies that reported outcomes that affect the user’s experience (eg, quality, satisfaction, efficiency, effectiveness without necessarily focusing on methodology), (4) studies carried out between 2006 and 2016, (5) studies published in English, and (6) type of study (any study design that is appropriate).

Exclusion criteria included (1) studies that focus on evaluations using solely experts and are not transferrable to user evaluations; (2) studies that are in the form of electronic book or are not freely available on the Web or through OpenAthens, the University of Bath library, or the University of the West of England library; (3)studies that evaluate banking, electronic commerce (e-commerce), or online libraries’ websites and do not have transferrable measures to a range of other websites; (4) studies that report exclusively on minority or special needs groups (eg, blind or deaf users); and (5) studies that do not meet all the inclusion criteria.

### Charting the Data

The next stage involved *charting* key items of information obtained from studies being reviewed. *Charting* [17] describes a technique for synthesizing and interpreting qualitative data by sifting, charting, and sorting material according to key issues and themes. This is similar to a systematic review in which the process is called data extraction. The data extracted included general information about the study and specific information relating to, for instance, the study population or target, the type of intervention, outcome measures employed, and the study design.

The information of interest included the following: type of website, aim or purpose of the study, study populations (users and experts), sample size, setting (laboratory, real life, and remotely assessed), website attributes evaluated, process of methodology, and process of analysis.

NVivo version 10.0 software was used for this stage by 2 researchers (RA and CH) to chart the data.

### Collating, Summarizing, and Reporting the Results

Although the scoping study does not seek to assess the quality of evidence, it does present an overview of all material reviewed with a narrative account of findings.

### Ethics Approval and Consent to Participate

As no primary research was carried out, no ethical approval was required to undertake this scoping study. No specific reference was made to any of the participants in the individual studies, nor does this study infringe on their rights in any way.

## Results

### Study Selection

**Figure 1:**
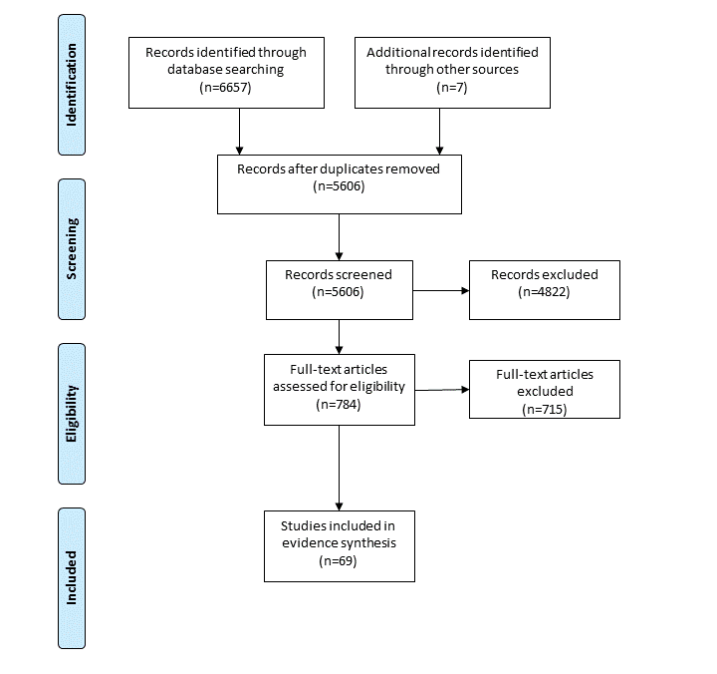
Preferred Reporting Items for Systematic Reviews and Meta-Analyses flowchart of search results.

The electronic database searches produced 6657 papers; a further 7 papers were identified through other sources. After removing duplicates (n=1058), 5606 publications remained. After titles and abstracts were examined, 784 full-text papers were read and assessed further for eligibility. Of those, 69 articles were identified as suitable by meeting all the inclusion criteria (Figure 1).

### Study Characteristics

#### Population

Studies referred to or used a mixture of users (72%) and experts (39%) to evaluate their websites; 54% used a controlled environment, and 26% evaluated websites remotely (Multimedia Appendix 1 [2-4,11,18-85]). Remote usability, in its most basic form, involves working with participants who are not in the same physical location as the researcher, employing techniques such as live screen sharing or questionnaires. Advantages to remote website evaluations include the ability to evaluate using a larger number of participants as travel time and costs are not a factor, and participants are able to partake at a time that is appropriate to them, increasing the likelihood of participation and the possibility of a greater diversity of participants [18]. However, the disadvantages of remote website evaluations, in comparison with a controlled setting, are that system performance, network traffic, and the participant’s computer setup can all affect the results.

A variety of types of websites evaluated were included in this review including government (9%), online news (6%), education (1%), university (12%), and sports organizations (4%). The aspects of quality considered, and their relative importance varied according to the type of website and the goals to be achieved by the users. For example, criteria such as ease of paying or security are not very important to educational websites, whereas they are especially important for online shopping. In this sense, much attention must be paid when evaluating the quality of a website, establishing a speciﬁc context of use and purpose [19].

The context of the participants was also discussed, in relation to the generalizability of results. For example, when evaluations used potential or current users of their website, it was important that computer literacy was reflective of all users [20]. This could mean ensuring that participants with a range of computer abilities and experiences were used so that results were not biased to the most or least experienced users.

#### Intervention

A total of 43 evaluation methodologies were identified in the 69 studies in this review. Most of them were variations of similar methodologies, and a brief description of each is provided in Multimedia Appendix 2. Multimedia Appendix 3 shows the methods used or described in each study.

#### Questionnaire

Use of questionnaires was the most common methodology referred to (37/69, 54%), including questions to rank or rate attributes and open questions to allow text feedback and suggested improvements. Questionnaires were used in a combination of before or after usability testing to assess usability and overall user experience.

#### Observed Browsing the Website

Browsing the website using a form of task completion with the participant, such as cognitive walkthrough, was used in 33/69 studies (48%), whereby an expert evaluator used a detailed procedure to simulate task execution and browse all particular solution paths, examining each action while determining if expected user’s goals and memory content would lead to choosing a correct option [30]. Screen capture was often used (n=6) to record participants’ navigation through the website, and eye tracking was used (n=7) to assess where the eye focuses on each page or the motion of the eye as an individual views a Web page. The think-aloud protocol was used (n=10) to encourage users to express out loud what they were looking at, thinking, doing, and feeling, as they performed tasks. This allows observers to see and understand the cognitive processes associated with task completion. Recording the time to complete tasks (n=6) and mouse movement or clicks (n=8) were used to assess the efficiency of the websites.

#### Qualitative Data Collection

Several forms of qualitative data collection were used in 27/69 studies (39%). Observed browsing, interviews, and focus groups were used either before or after the use of the website. Pre-website-use, qualitative research was often used to collect details of which website attributes were important for participants or what weighting participants would give to each attribute. Postevaluation, qualitative techniques were used to collate feedback on the quality of the website and any suggestions for improvements.

#### Automated Usability Evaluation Software

In 9/69 studies (13%), automated usability evaluation focused on developing software, tools, and techniques to speed evaluation (rapid), tools that reach a wider audience for usability testing (remote), and tools that have built-in analyses features (automated). The latter can involve assessing server logs, website coding, and simulations of user experience to assess usability [42].

#### Card Sorting

A technique that is often linked with assessing navigability of a website, card sorting, is useful for discovering the logical structure of an unsorted list of statements or ideas by exploring how people group items and structures that maximize the probability of users finding items (5/69 studies, 7%). This can assist with determining effective website structure.

#### Web Usage Analysis

Of 69 studies, 3 studies used Web usage analysis or Web analytics to identify browsing patterns by analyzing the participants’ navigational behavior. This could include tracking at the widget level, that is, combining knowledge of the mouse coordinates with elements such as buttons and links, with the layout of the HTML pages, enabling complete tracking of all user activity.

#### Outcomes (Attributes Used to Evaluate Websites)

Often, different terminology for website attributes was used to describe the same or similar concepts (Multimedia Appendix 4). The most used website attributes that were assessed can be broken down into 8 broad categories and further subcategories:

1. Usability or ease of use is the degree to which a website can be used to achieve given goals (n=58). It includes navigation such as intuitiveness, learnability, memorability, and information architecture; effectiveness such as errors; and efficiency.
2. Content (n=41) includes completeness, accuracy, relevancy, timeliness, and understandability of the information.
3. Web design criteria (n=29) include use of media, search engines, help resources, originality of the website, site map, user interface, multilanguage, and maintainability.
4. Functionality (n=31) includes links, website speed, security, and compatibility with devices and browsers.
5. Appearance (n=26) includes layout, font, colors, and page length.
6. Interactivity (n=25) includes sense of community, such as ability to leave feedback and comments and email or share with a friend option or forum discussion boards; personalization; help options such as frequently answered questions or customer services; and background music.
7. Satisfaction (n=26) includes usefulness, entertainment, look and feel, and pleasure.
8. Loyalty (n=8) includes first impression of the website.

## Discussion

### GoodWeb: Website Evaluation Guide

**Figure 2:**
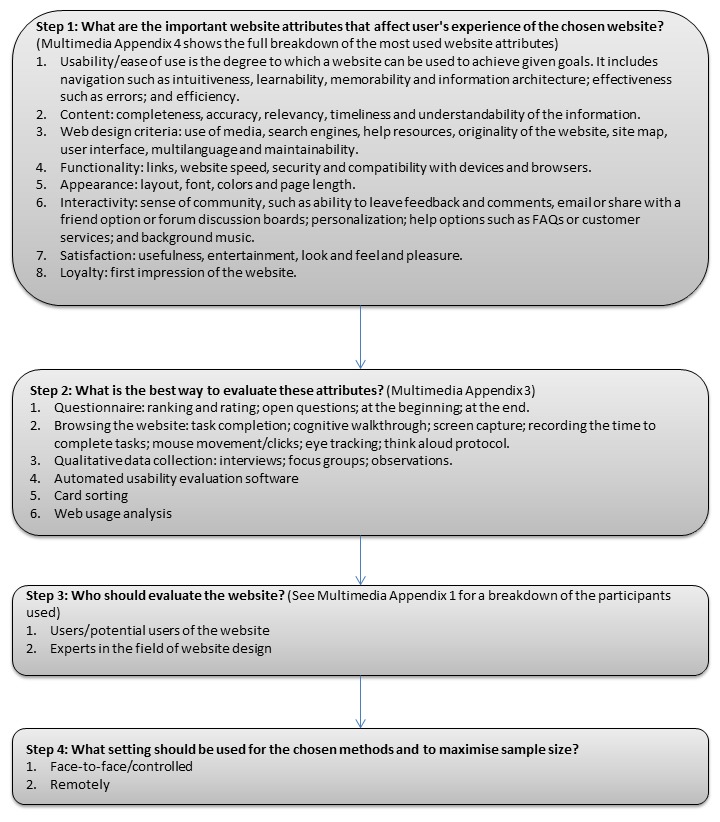
Framework for website evaluation.

As there was such a range of methods used, a suggested guide of options for evaluating websites is presented below (Figure 2), coined GoodWeb, and applied to an evaluation of e-Bug, an international educational health website [14]. Allison at al [86] show the full details of how GoodWeb has been applied and outcomes of the e-Bug website evaluation.

#### Step 1. What Are the Important Website Attributes That Affect User's Experience of the Chosen Website?

Usability or ease of use, content, Web design criteria, functionality, appearance, interactivity, satisfaction, and loyalty were the umbrella terms that encompassed the website attributes identified or evaluated in the 69 studies in this scoping study. Multimedia Appendix 4 contains a summary of the most used website attributes that have been assessed. Recent website evaluations have shifted focus from usability of websites to an overall user’s experience of website use. A decision on which website attributes to evaluate for specific websites could come from interviews or focus groups with users or experts or a literature search of attributes used in similar evaluations.

#### Application

In the scenario of evaluating e-Bug or similar educational health websites, the attributes chosen to assess could be the following:

1. Appearance: colors, fonts, media or graphics, page length, style consistency, and first impression
2. Content: clarity, completeness, current and timely information, relevance, reliability, and uniqueness
3. Interactivity: sense of community and modern features
4. Ease of use: home page indication, navigation, guidance, and multilanguage support
5. Technical adequacy: compatibility with other devices, load time, valid links, and limited use of special plug-ins
6. Satisfaction: loyalty

These cover the main website attributes appropriate for an educational health website. If the website did not currently have features such as search engines, site map, background music, it may not be appropriate to evaluate these, but may be better suited to question whether they would be suitable additions to the website; or these could be combined under the heading *modern features*. Furthermore, security may not be a necessary attribute to evaluate if participant identifiable information or bank details are not needed to use the website.

#### Step 2. What Is the Best Way to Evaluate These Attributes?

Often, a combination of methods is suitable to evaluate a website, as 1 method may not be appropriate to assess all attributes of interest [29] (see Multimedia Appendix 3 for a summary of the most used methods for evaluating websites). For example, screen capture of task completion may be appropriate to assess the efficiency of a website but would not be the chosen method to assess loyalty. A questionnaire or qualitative interview may be more appropriate for this attribute.

#### Application

In the scenario of evaluating e-Bug, a questionnaire before browsing the website would be appropriate to rank the importance of the selected website attributes, chosen in step 1. It would then be appropriate to observe browsing of the website, collecting data on completion of typical task scenarios, using the screen capture function for future reference. This method could be used to evaluate the effectiveness (number of tasks successfully completed), efficiency (whether the most direct route through the website was used to complete the task), and learnability (whether task completion is more efficient or effective second time of trying). It may then be suitable to use a follow-up questionnaire to rate e-Bug against the website attributes previously ranked. The attribute ranking and rating could then be combined to indicate where the website performs well and areas for improvement.

#### Step 3: Who Should Evaluate the Website?

Both users and experts can be used to evaluate websites. Experts are able to identify areas for improvements, in relation to usability; whereas, users are able to appraise quality as well as identify areas for improvement. In this respect, users are able to fully evaluate user’s experience, where experts may not be able to.

#### Application

For this reason, it may be more appropriate to use current or potential users of the website for the scenario of evaluating e-Bug.

#### Step 4: What Setting Should Be Used?

A combination of controlled and remote settings can be used, depending on the methods chosen. For example, it may be appropriate to collect data via a questionnaire, remotely, to increase sample size and reach a more diverse audience, whereas a controlled setting may be more appropriate for task completion using eye-tracking methods.

### Strengths and Limitations

A scoping study differs from a systematic review, in that it does not critically appraise the quality of the studies before extracting or *charting* the data. Therefore, this study cannot compare the effectiveness of the different methods or methodologies in evaluating the website attributes. However, what it does do is review and summarize a huge amount of literature, from different sources, in a format that is understandable and informative for future designs of website evaluations.

Furthermore, studies that evaluate banking, e-commerce, or online libraries’ websites and do not have transferrable measures to a range of other websites were excluded from this study. This decision was made to limit the number of studies that met the remaining inclusion criteria, and it was deemed that the website attributes for these websites would be too specialist and not necessarily transferable to a range of websites. Therefore, the findings of this study may not be generalizable to all types of website. However, Multimedia Appendix 1 shows that data were extracted from a very broad range of websites when it was deemed that the information was transferrable to a range of other websites.

A robust website evaluation can identify areas for improvement to both fulfill the goals and desires of its users [62] and influence their perception of the organization and overall quality of resources [48]. An improved website could attract and retain more online users; therefore, an evidence-based website evaluation guide is essential.

### Conclusions

This scoping study emphasizes the fact that the debate about how to define the quality of websites remains open, and there are numerous approaches and models to evaluate it. Multimedia Appendix 2 shows existing methodologies or tools that can be used to evaluate websites. Many of these are variations of similar approaches; therefore, it is not strictly necessary to use these tools at face value; however, some could be used to guide analysis, following data collection. By following steps 1 to 4 of GoodWeb, the framework suggested in this study, taking into account the desired participants and setting and website evaluation methods, can be tailored to the needs of specific websites and individual aims of evaluations.

## Appendix

Multimedia Appendix 1 Summary of included studies, including information on the participant.

Multimedia Appendix 2 Interventions: methodologies and tools to evaluate websites.

Multimedia Appendix 3 Methods used or described in each study.

Multimedia Appendix 4 Summary of the most used website attributes evaluated.

## Abbreviations

E-commerce: electronic commerce
