# Supplementary material for: A Comprehensive Framework to Evaluate Websites: Literature Review and Development of GoodWeb
Source: JMIR Form Res. 2019 Oct 24;3(4):e14372. doi: 10.2196/14372 (PMC6914275; doi:10.2196/14372)
Supplement: Multimedia Appendix 1 [file formative_v3i4e14372_app1.pdf]

Multimedia Appendix 1. Summary of included studies, including information on the participants

Title (Citation)	Type of website (industry/category)	Aim/purpose of the study	Study participants (sample size)	Setting[1]
Usability Evaluation Basics **("Usability Evaluation Basics,")**	NA	To explore the basics of usability evaluation	Users	NA
10 Criteria for Better Website Usability: Heuristics Cheat Sheet **("10 Criteria for Better Website Usability: Heuristics Cheat Sheet,")**	NA	To list ten questions you can ask to evaluate your own website	Experts	NA
Quality evaluation of E-government websites of Turkey **(Akg, 2016)**	Government	To assess the Turkish E-government websites in term to their usability and accessibility.	Expert (n=1)	NA
Ranking quality factors for measuring web service quality **(Al Zaghoul, Al Nsour, & Rababah, 2010)**	NA	This paper defines and categorizes the quality factors for measuring web service quality. Moreover, the paper includes a framework to establish quality factors in terms of attributes, in addition to their level of importance based on the opinion of highly-skilled professionals. The primary goals are identifying, qualifying, categorizing and ranking these factors. These factors would be used to give indications for the improvement of the web services quality.	Experts	Remote
Users' performance in lab and non-lab environments through online usability testing: A case of evaluating the usability of digital academic libraries' websites **(Alharbi & Mayhew, 2015)**	Digital libraries	A. To investigate the effectiveness of online usability studies in providing data on the test performance<br>B. To investigate the differences in user performance between online testing in labs versus natural environments	Users (n=30)	Controlled and remote
Preliminary investigation of Islamic websites design & content feature: A heuristic evaluation from user perspective **(Aliyu, Mahmud, & Md. Tap, 2010)**	Islamic websites	To investigate the quality ranking of current Islamic websites using nine (9) website features (i.e. navigation, interactivity, legitimacy, objectivity, authority, relevancy, attractiveness, credibility, and reliability) in order to find the highest and lowest quality scores for individual websites based on their design & content feature.	Expert (n=1)	NA
Evaluating design features of Islamic websites: A Muslim user perception **(Aliyu, Mahmud, Tap, & Nassr, 2013)**	Islamic websites	Identifying the most significant design features of Islamic websites that influence website use.	Users (89)	Remote
Usability evaluation of online news websites: A user perspective Approach **(Al-Radaideh, Abu-Shanab, Hamam, & Abu-Salem, 2011)**	Online news	To evaluate online newspaper websites using two assessment measures; usability and web content.	Users (n=204)	NA
Proposal of a tool of support to the evaluation of user in educative web sites **(Alva et al., 2008)**	Education	To present an analysis of the necessity to implement a tool of evaluation and measurement of the usability to support the ME-USitE methodology that contributes to the detection of problems and failures of usability from the perspective of the user of educative Web sites in a real work environment.	Users and experts	NA
Using think-aloud and psychometrics to explore users' experience with a news web site **(Gabor Aranyi, van Schaik, & Barker, 2012)**	Online news	To develop and test a psychological model of end-users' experience with news sites.	Users (n=77)	Controlled

[1] The setting refers to the location and environment in which the study was carried out in. 'Controlled' refers to laboratory setting in which external stimuli are removed; 'remote' refers to the subject participating in the study from an uncontrolled setting eg. following a link on an email, 'NA' either means that it is not applicable eg if it is a literature review, or that the study does not make reference to the setting

Interdependence between technical web accessibility and usability: Its influence on web quality models **(M. Arrue, Fajardo, López, & Vigo, 2007)**	Mixture: general information magazine, online shop, gastronomic, university, corporative, institutional websites, etc.	To determine whether diverse accessibility and usability metrics correlate. Concretely, our hypothesis was that the more accessible websites were measured in terms of the POUR metrics (M Arrue, Vigo, & Abascal, 2005), the more usable they would be for general users in terms of target found, response time, disorientation and satisfaction.	Users and experts	NA
Knowing the user's every move: User activity tracking for website usability evaluation and implicit interaction **(Atterer, Wnuk, & Schmidt, 2006)**	NA	To describe our requirements for user tracking and the chosen approach<br>To discusses the types of data that can be obtained using activity tracking as well as its use for implicit interaction and usability testing<br>To explain the implementation of our HTTP proxy for activity tracking is explained	Users (n=12)	Controlled
Website evaluation measures, website user engagement and website credibility for Municipal website **(Bahry, Masrom, & Masrek, 2015)**	Government	To explore website evaluation measures specifically for information driven websites, towards website credibility and user engagement.	Users	NA
Investigating factors affecting students' satisfaction of university websites **(Bairamzadeh & Bolhari, 2010)**	University websites	To propose universities website satisfaction model	Users (n=270)	Remote
Improving user experience: A methodology proposal for web usability measurement **(Bañón-Gomis, Tomás-Miquel, & Expósito-Langa, 2014)**	NA	To analyse, and improve on, currently available tools for measuring web usability, and propose a new evaluation methodology which will reduce the uncertainty that is inherent in the tools that currently exist	NA	NA
Data triangulation and web quality metrics: A case study in e-government **(Barnes & Vidgen, 2006)**	Government	To evaluate the quality perceptions of users of an electronic government website	Users (n=420)	Remote
Quality of Web usability evaluation methods: An empirical study on MiLE+ **(Bolchini & Garzotto, 2007)**	Museum of Art website	To investigate the concepts of quality and quality measurement for web usability evaluation methods, aiming to raise a critical reflection on these issues.	Experts (n=42)	Controlled
Evaluating the enhancement of corporate social responsibility websites quality based on a new hybrid MADM model **(Chen, Tzeng, & Chang, 2015)**	Corporate Social Responsibility (CSR) websites	To analyse websites of benchmark companies for establishing an evaluation model to be a reference for CSR website design	Experts	NA
An exploratory study on websites quality assessment **(Cherfi, Tuan, & Comyn-Wattiau, 2014)**	NA	To check whether the main metrics proposed by researchers can be mapped towards ISO 9126 quality sub-characteristics and how this map- ping covers the six main characteristics.	Users and experts	NA
A hybrid fuzzy MCDM approach for evaluating website quality of professional accounting firms	CPA firm websites	To build a hybrid approach that combines the fuzzy analytic network process (FANP) and fuzzy VlseKriterijumska Optimizacija I	NA	NA

**(Chou & Cheng, 2012)**		Kompromisno Resenje (FVIKOR) for evaluating website quality of the top-four CPA firms in Taiwan and provide worth- while recommendations for enhancing website design and content.		
An Introduction to Website Usability Testing **(Churm, 2012)**	NA	To give an overview of the 3 main categories of usability testing:<br>Explorative: Used early in product development to assess the effectiveness and usability of a preliminary design or prototype, as well as users' thought processes and conceptual understanding.<br>Assessment: Used midway in product development or as an overall usability test for technology evaluation. Evaluates real-time trials of the technology to determine the satisfaction, effectiveness, and overall usability.<br>Comparative: Compares two or more instructional technology products or designs and distinguishes the strengths and weaknesses of each.	Users and experts	Controlled and remote
Evaluation of Quality, Content, and Use of the Web Site Prepared for Family Members Giving Care to Stroke Patients **(Demir & Gozum, 2015)**	Health	To evaluate the quality, content, usability, and efficacy of a Web site pre- pared for the purpose of improving the caregiving capability of family members who provide care for stroke survivors at home.	Users (n=38); experts (n=10)	Controlled
University website quality comparison by using non-parametric statistical test: A case study from Malaysia **(Dominic, Jati, & Hanim, 2013)**	University websites	1. to propose the new methodology for evaluating the quality of Malaysian University websites<br>2. to determine the best Malaysian University website based on the criteria proposed in the new methodology<br>3. to determine the best ranking method used to evaluate website quality.	Users and experts	NA
Measuring the quality of governmental websites in a controlled versus an online setting with the 'Website Evaluation Questionnaire' **(Elling, Lentz, de Jong, & van den Bergh, 2012)**	Government	To analyse the underlying factor structure, the stability and reliability of this structure, and the sensitivity of the WEQ (Website Evaluation Questionnaire) to quality differences between websites.	Users (n=1858)	Controlled (n=273) and remote (n=1585)
Impacts of navigation structure, task complexity, and users' domain knowledge on Web site usability-an empirical study **(Fang & Holsapple, 2011)**	Quality management, production planning and scheduling, facility management, inventory management, and project management websites.	For a given sponsor trying to facilitate the KA (knowledge acquisition) activities of users engaged in certain kinds of tasks and operating within a given environment, what system features can be designed into a Web site to enhance its usability? This study aims to answer this question with respect to alternative navigation structures that can be designed into a Web site.	Users (n=178); experts (n=134)	Controlled
A systematic review on the effectiveness of web usability evaluation methods **(Fernandez,**	NA	Which usability evaluation methods have proven to be the most effective in the Web domain?	Users and experts	Controlled and remote

**Abrahão, & Insfran, 2012)**				
Evaluating web site quality: The value of a multi paradigm approach **(Fink & Nyaga, 2009)**	Public accounting (PA) firms' websites	To benchmark the quality of web sites of major public accounting (PA) firms by seeking the opinions of potential clients and analysing the data to establish best quality practice for PA web sites.	Users (102)	Controlled
The role played by perceived usability, satisfaction and consumer trust on website loyalty **(Carlos Flavián, Miguel Guinalíu, & Raquel Gurrea, 2006)**	NA	To determine the influence that perceived usability has on the user's loyalty to websites that they visit.	NA	NA
The influence of familiarity and usability on loyalty to online journalistic services: The role of user experience **(C. Flavián, M. Guinalíu, & R. Gurrea, 2006)**	Online news	To analyse the effect of consumer familiarity with a website and the degree of perceived usability on levels of loyalty.	NA	NA
Application of quality management tools in the evaluation of websites: The case of sports organizations **(Gonzalez, Quesada, Davis, & Mora-Monge, 2015)**	Sports organisation's website	To demonstrate how the WebQual instrument together with quality function deployment (QFD) and benchmarking analysis offer a more precise approach to examining design-side elements of website quality.	Users (n=1763)	Controlled
Deconstructing web experience: More than just usability and good design **(Harrison & Pétrie, 2007)**	NA	1. Are there trends in perceptions of aspects of web design that facilitate affective or cognitive responses relating to curiosity, creativity, challenge, surprise and an altered sense of time?<br>2. Are all of the aspects reported by respondents covered by usability or design guidelines?	Users (n=141)	Remote
Usability testing of web sites designed for communities of practice: tests of the IEEE Professional Communication Society (PCS) web site combining specialized heuristic evaluation and task-based user testing **(Hart & Portwood, 2009)**	IEEE PCS (Professional Communication Society) website	To describe a two-part methodology the authors developed for usability tests of the IEEE PCS Web site that combines heuristic evaluation and task- based testing.	Users and experts	Controlled and remote
Extracting usability and user experience information from online user reviews **(Hedegaard & Simonsen, 2013)**	NA	To investigate the content of online reviews with the aims of (i) charting the distribution of information in reviews among different dimensions of usability and UX, and (ii) extracting an associated vocabulary for each dimension using techniques from natural language processing and machine learning.	Experts (n=8)	Controlled
A multi-granular linguistic hierarchical model to evaluate the quality of web site services **(Herrera et al., 2006)**	NA	To propose a linguistic quality evaluation model to evaluate the services offered by the web sites.	Users	NA
Applying usability testing techniques to improve a health promotion website **(Hinchliffe & Mummery, 2008)**	Health	To employ usability testing of an existing health promotion website (www.10000steps.org.au) to inform modifications to the website and to identify common usability themes in the redevelopment of this site that may serve to guide future website development and maintenance in the field of health promotion.	Users (n=12); experts (n=3)	Controlled

Usability testing through websites **(Ijaz & Andlib, 2014)**	NA	Test the usability of websites	Users (n=34)	Controlled
Translating access into utilization: Lessons from the design and evaluation of a health insurance Web site to promote reproductive health care for young women in Massachusetts **(Janiak, Rhodes, & Foster, 2013)**	Health	To assess the health literacy demands and usability of the site among its target audience, women ages 18–26 years.	Users (n=8)	Controlled
Multi-attribute evaluation of website quality in e-business using an integrated fuzzy AHP-TOPSIS methodology **(Kaya, 2010)**	e-business websites	To propose a multi-attribute e-business website quality evaluation methodology based on a modified fuzzy TOPSIS approach.	Experts	NA
Measuring website quality: Asymmetric effect of user satisfaction **(Kincl & Štrach, 2012)**	University websites	To compare and contrast attributes of user satisfaction based on usability guidelines seeking to identify practical easy-to-administer measurement tools.	Users (n=30)	Controlled
Perceived website aesthetics by users and designers: Implications for evaluation practice **(Koutsabasis & Istikopoulou, 2014)**	Online sports news, paint company's websites	The goal of the evaluation study was twofold: first, to set up a practical approach for the evaluation of aesthetics of websites based on principles of the usability testing method and second to assess important dimensions of aesthetics of websites and investigate them with their designers.	Users (n=111); experts (n=3)	Controlled
Vertical versus dynamic menus on the world wide web: Eye tracking study measuring the influence of menu design and task complexity on user performance and subjective preference **(Leuthold, Schmutz, Bargas-Avila, Tuch, & Opwis, 2011)**	e-commerce	To compare the influence of different navigation designs (vertical versus dynamic menus) and task complexity (simple versus complex navigation tasks) on user performance, navigation strategy, and subjective preference.	Users (n=120)	Controlled
Evaluating Website Quality: Applying Cue Utilization Theory to WebQual **(Longstreet, 2010)**	NA	To answer the question, which informational cues (i.e., website characteristics) are the most influential when consumers make an evaluation of overall website quality? Specifically, how do consumers perceptions of the value of informational cues and their confidence in rating informational cues influence their perception of a website's quality?	Users (n=500)	Remote
Measuring user experience of usability tool, designed for higher educational websites **(Manzoor, 2013)**	University websites	To measure the user experience of software, designed for the usability evaluation of the higher educational websites in order to enhance the usability.	Experts (n=21)	Remote
Application of fuzzy analytic hierarchy process to evaluate the quality of e-government web sites **(Markaki et al., 2010)**	Government	To adopt Fuzzy Analytic Hierarchy Process, a fuzzy multiple attribute decision making method, to assess the quality attributes of e-government websites.	NA	NA
22 Essential Tools for Testing Your Website's Usability **(Mashable)**	NA	To discuss six crucial factors that affect usability	Users	Remote
Web usability: Principles and evaluation methods **(Matera, Rizzo, & Carughi, 2006)**	NA	To introduce principles and evaluation methods to be adopted during the whole application lifecycle for promoting usability.	Users and experts	Controlled and remote

Designing an educational website to improve quality of supportive oncology care for women with ovarian cancer: An expert usability review and analysis **(McClellan et al., 2016)**	Health	To focus on the preliminary usability review conducted, subsequent changes, and final usability evaluation.	Users	Controlled
A quality evaluation methodology for health-related websites based on a 2-tuple fuzzy linguistic approach **(Moreno et al., 2010)**	Health	To present a qualitative and user-oriented methodology for assessing quality of health- related websites based on a 2-tuple fuzzy linguistic approach.	Users	Controlled
Detecting low usability web pages using quantitative data of users' behaviour **(Nakamichi, Shima, Sakai, & Matsumoto, 2006)**	NA	To detect low usability web pages from the behaviour of users, such as browsing time, mouse movement and eye movement.	Users (n=10)	Controlled
An empirical study of factors affecting the perceived usability of websites for student Internet users **(Nathan & Yeow, 2009)**	Mixture	• To determine the crucial Web usability factors from the perspective of Malaysian SIUs (Student Internet Users).<br>• To determine if SIUs' demographics (e.g., race and gender) affect their perception of Web usability.<br>• To identify elements in websites that affect the Web usability factors.<br>• To recommend specific usability guidelines for designing websites targeting SIUs.	Users	Controlled
A user-centred evaluation framework for the Sealife semantic web browsers **(Oliver et al., 2009)**	Digital libraries	To describe a user-centred evaluation framework that was developed to evaluate the Sealife SWBs that elicited feedback on users' perceptions on ease of use and information findability.	Users (85)	Controlled and remote
An experience with measuring multi-user online task performance **(Paul, Yadamsuren, & Erdelez, 2012)**	University websites	To introduce a new approach to conducting MUST using the Autopilot feature of Morae usability evaluation software as a method for discounted usability testing on an academic website.	Users (n=17)	Controlled
What do users really care about? A comparison of usability problems found by users and experts on highly interactive websites **(Petrie & Power, 2012)**	Government	To investigate the usability problems found in the evaluation of six highly interactive websites by 30 users in a task-based evaluation and 14 experts using three different expert evaluation methods.	Users (n=15); experts (n=3)	Controlled
Fuzzy reduced method for evaluating the quality of institutional web sites **(Rekik & Kallel, 2011)**	University websites	To present a quality assessment method and model (Fuzz-web) in order to measure the performance of dynamic institutional websites.	Users	NA
The secret to patron-centered Web design: cheap, easy, and powerful usability techniques **(Reynolds, 2008)**	NA	To describe cheap, easy, and powerful usability techniques.	Users	Controlled
Eyes don't lie: Understanding users' first impressions on websites using eye tracking **(Sheng, Lockwood, & Dahal, 2013)**	University websites	To examine the amount of exposure time needed to form first impression<br>To identify the web design factors that influence the formation of a users' first impression<br>To study the emotional responses of users on website design<br>To understand the relationship between first impression and eye	Users (n=20)	Controlled

		movement.		
Measuring web-based service quality: The online customer point of view **(Swaid & Wigand, 2007)**	NA	To propose and tests a conceptual framework of web-based service quality by using the SERVQUAL measure as its starting point<br>To examine the influence of identified dimensions of web-based service quality on overall service quality and loyalty intentions.	Users	Remote
An empirical study of Web browsing behaviour: Towards an effective Website design **(G. W. Tan & Wei, 2006)**	Dell's website	To build a conceptual model of an effective website design that aimed to reduce cognitive overhead involved and facilitate website browsing in its optimal manner; as well as formation of guidelines to assist designers to better identify areas for improvement and create effective website.	Users (n=6)	NA
Web evaluation: Heuristic evaluation vs. user testing **(W. s. Tan, Liu, & Bishu, 2009)**	Mixture	To compare the efficiency and effectiveness between user testing and heuristic analysis in evaluating four different commercial web sites.	Users (n=12); experts (n=9)	Controlled
Consumer perspectives on quality attributes in evaluating health websites **(Tao, LeRouge, Deckard, & De Leo, 2011)**	Health	1) provide a comprehensive listing of quality attributes deemed relevant by general healthcare consumers<br>2) establish the importance of specific quality attributes in order to improve content and design based on the input of general healthcare consumers<br>3) examine differences in perceptions of various quality attributes between health consumers with healthcare background and those with specified backgrounds in another field (business in this case)	Users (n=198)	Controlled
Conducting a Quick and Dirty Evaluation of your Website's Usability **(The Whole Brain Group, 2011)**	NA	To show you how you can complete your own usability evaluation in a much more affordable (and quick!) way using a heuristic evaluation.	Experts (n=3-5)	Controlled
User evaluation of websites: From first impression to recommendation **(Thielsch, Blotenberg, & Jaron, 2014)**	Mixture, including: corporate websites, e- commerce, e-recruiting, entertainment and information sites.	To further explore and analyse the interplay of content, usability and aesthetics in evaluations of websites given by website users.	Users (n=330)	Controlled
Web site usability with remote users: Formal usability studies and focus groups **(Thomsett-Scott, 2006)**	Digital libraries	The purpose of this paper is to provide an overview of how the traditional usability techniques of focus groups and formal usability studies can be extended to studies involving off-campus users.	Users	Remotely
An effective evaluation model and improvement analysis for national park websites: A case study of Taiwan **(Tsai et al., 2010)**	National parks' websites	1) provides a comprehensive and systematic approach that quantitatively measures a website's overall performance<br>2) contributes to practical applications in terms of providing worthwhile recommendations for building an ideal website.	Experts (n=16)	NA

Attributes of web site usability: A study of web users with the repertory grid technique **(Tung, Xu, & Tan, 2009)**	B2C sites (business that sells products or provides services to end-user consumers)	The present study, building on these prior studies (Agarwal & Venkatesh; Venkatesh & Ramesh, 2006), is particularly interested in ascertaining Web users' perspectives on the attributes they consider important to B2C Web sites and in providing a richer understanding of the meanings Web users ascribe to the MUG attributes, which can then be used to appraise and enhance the MUG.	Users (n=25)	Controlled
Development of evaluation heuristics for web service user experience **(Vaananen-Vainio-Mattila & Wäljas, 2009)**	Facebook<br>Nokia Sports Tracker<br>TripAdvisor	The goal of our research is to understand the characteristics of Web service UX (user experience) and to develop tools for HCI practitioners for UX design and evaluation.	Experts (n=3)	NA
Examining the impacts of website complexities on user satisfaction based on the task-technology fit model: An experimental research using an eyetracking device **(Wang, Wang, & Wei, 2014)**	100 most popular websites in Taiwan	To get insights into how Internet users perceive the quality and user-friendliness of website design, this study intends to conduct a controlled experiment using an eye-tracking device to examine the effects of different levels of the three primary features of website complexity, namely the component, coordinative, and dynamic complexities on the satisfaction and intentions to reuse of website users. Additionally, in conjunction with the concept of website complexities, the task-technology fit theory (TTF) is adopted to develop a research framework to understand the behaviours of the website users.	Users (n=120)	Controlled
Toward an analytical approach for effective Web site design: A framework for modeling, evaluation and enhancement **(B. Yen, Hu, & Wang, 2007)**	NA	To propose a framework which classifies real-world design problems into generic website design categories and maps each resulting category into a graph model which can be analysable or solved using appropriate analytical techniques.	NA	NA
A comparison of usability evaluation methods: heuristic evaluation versus end-user think-aloud protocol - an example from a web-based communication tool for nurse scheduling **(P. Y. Yen & Bakken, 2009)**	Two Bidshift interfaces: Nurse Manager Interface (NMI) and Staff Nurse Interface (SNI)	To compare the results from HCI heuristic evaluation and end-user think-aloud protocol.	Experts (n=5)	Controlled
