# Supplementary material for: A Comprehensive Framework to Evaluate Websites: Literature Review and Development of GoodWeb
Source: JMIR Form Res. 2019 Oct 24;3(4):e14372. doi: 10.2196/14372 (PMC6914275; doi:10.2196/14372)
Supplement: Multimedia Appendix 2 [file formative_v3i4e14372_app2.pdf]

Multimedia Appendix 2. Interventions: Methodologies and tools to evaluate websites

Evaluation methodology/tool	Studies referring/using the methodology /tool	Brief description of the methodology/tool
1. 2QCV3Q model	(M. Arrue, Fajardo, López, & Vigo, 2007)<br>(Cherfi, Tuan, & Comyn-Wattiau, 2014)<br>(Rekik & Kallel, 2011)	Evaluates website quality based on seven dimensions: who, what, why, when, where, how, and with what means and devices.
2. Analytical Hierarchy Process	(Cherfi, Tuan, & Comyn-Wattiau, 2014)<br>(Dominic, Jati, & Hanim, 2013)<br>(Kaya, 2010)<br>(Markaki, Charilas, & Askounis, 2010)<br>(Tsai, Chou, & Lai, 2010)	Analytical hierarchy process (AHP) is a popular model to aggregate multiple criteria for decision-making.
3. DEMATEL-Based Analytic Network Process (DANP)	(Chen, Tzeng, & Chang, 2015)	DEMATEL-Based Analytic Network Process (DANP), a novel combination of the DEMATEL and Analytic Network Process (ANP) methods were adopted to calculate the weights of the criteria.
4. Delphi method	(Al Zaghoul, Al Nsour, & Rababah, 2010)	A multistage process designed to combine individual opinion into group consensus. The method requires knowledgeable and expert contributors individually responding to questions about the website and submitting the results to a central coordinator. The coordinator processes the contributions and feeds the results back to the respondents. The respondents are then asked to resubmit their views, assisted by the input provided by the coordinator. This process continues until the coordinator sees that a consensus has formed. The technique was intended to remove the bias that is possible when diverse groups of experts meet together. In the Delphi technique, the experts do not know who the other experts are during the process.
5. Decision-Making Trial and Evaluation Laboratory (DEMATEL)	(Chen, Tzeng, & Chang, 2015)<br>(Tsai, Chou, & Lai, 2010)	Decision-Making Trial and Evaluation Laboratory (DEMATEL) method deals with the interdependence between evaluation criteria and converts the criteria's cause and effect relations into a visual structural map.
6. DISCERN	(Demir & Gozum, 2015)	This instrument evaluates the quality of training materials providing written information about the treatment options for health problems. The total score on the 16 items ranges from 15 to 75.Each item is rated from 1 to 5. A 16th item, which provides general assessment, is evaluated separately. Whereas low DISCERN scores show that the quality is poor, high scores show a good quality.

7. Diagnostic Recorder for Usability Measurement (DRUM)	(Alva et al., 2008)	Diagnostic Recorder for Usability Measurement (DRUM), a software tool for video-assisted usability evaluation, helps evaluators to organise and analyse user-based evaluations, and to deliver measures and diagnostic data.
8. E-S-QUAL	(Tsai, Chou, & Lai, 2010)	A 22-item scale of four dimensions: efficiency, fulfilment, system availability, and privacy, used to assessing electronic service quality.
9. Fuzzy Analytical Hierarchy Process (FAHP)	(Dominic, Jati, & Hanim, 2013)<br>(Markaki, Charilas, & Askounis, 2010)<br>(Rekik & Kallel, 2011)	Fuzzy Analytical Hierarchy Process (FAHP), a fuzzy extension of AHP, can be used to solve hierarchical fuzzy problems.
10. Fuzzy analytic network process (FANP)	(Chou & Cheng, 2012)	Fuzzy analytic network process (FANP) links fuzzy concepts with network analysis process. This method can be useful when the decision faced with several options and decision indicators. The theory of fuzzy system through using fuzzy logic theory and fuzzy sizes can enter parameters such as knowledge, experience and human judgment, in to the model.
11. Fuzzweb	(Rekik & Kallel, 2011)	An expert- based methodology based on a benchmark of institutional websites. It involves: the user selecting and evaluating criteria for a website with the evaluation tools; use of fuzzy computation; and ranking of the website.
12. Fuzzy linguistic approach	(Herrera et al., 2006)<br>(Moreno, Morales del Castillo, Porcel, & Herrera-Viedma, 2010)	The fuzzy linguistic approach is an approximate technique, which represents qualitative aspects as linguistic values by means of linguistic variables, that is, variables whose values are not numbers but words or sentences in a natural or artificial language.
13. Fuzzy VlseKriterijumska Optimizacija I Kompromisno Resenje (FVIKOR)	(Chou & Cheng, 2012)<br>(Tsai, Chou, & Lai, 2010)	Fuzzy VlseKriterijumska Optimizacija I Kompromisno Resenje (FVIKOR) is an algorithm used to evaluate websites' quality and rank the order among them.
14. Heuristic evaluation	("10 Criteria for Better Website Usability: Heuristics Cheat Sheet,")<br>(Aliyu, Mahmud, & Md. Tap, 2010)<br>(Al-Radaideh, Abu-Shanab, Hamam, & Abu-Salem, 2011)<br>(M. Arrue, Fajardo, López, & Vigo, 2007)<br>(Bañón-Gomis, Tomás-Miquel, & Expósito-Langa, 2014)<br>(Hart & Portwood, 2009)<br>(Elling, Lentz, de Jong, & van den Bergh, 2012)<br>(Fernandez, Abrahão, &	Heuristic evaluation is one of the most common techniques, whereby HCI (human-computer interaction) experts discover system usability problems by detecting unmet criteria (heuristic principles) i.e. heuristic violations.

	Insfran, 2012)<br>(Matera, Rizzo, & Carughi, 2006)<br>(Nathan & Yeow, 2009)<br>(Petrie & Power, 2012)<br>(W. s. Tan, Liu, & Bishu, 2009)<br>(Tao, LeRouge, Deckard, & De Leo, 2011)<br>(The Whole Brain Group, 2011)<br>(Thielsch, Blotenberg, & Jaron, 2014)<br>(Thomsett-Scott, 2006)<br>(P. Y. Yen & Bakken, 2009)	
15. Link Popularity	(Cherfi, Tuan, & Comyn-Wattiau, 2014)	Link Popularity measures the total number of websites that link to this site.
16. Linear weightage model (LWM)	(Dominic, Jati, & Hanim, 2013)<br>(Rekik & Kallel, 2011)	Linear weightage model (LWM) is an evaluation method whereby users have to assign weights to the website criteria. In most cases, there are some criteria considered as more important than others, such as load time, response time, traffic, page rank and broken link. Decision-makers should assign weight to each individual criterion to determine the relative importance of each one.
17. ME-USitE	(Alva et al., 2008)	ME-USitE is a methodology used to measure and evaluate the usability of educative websites. The approach tries to complement the evaluation from the perspective of the user, using the method of investigation; and from the perspective of the expert, using inspection methods.
18. Milano Lugano Evaluation Method – version 2 (MiLE+)	(Bolchini & Garzotto, 2007)	MiLE+ (Milano Lugano Evaluation Method – version 2) is the evolution of two previous inspection techniques for the usability of hypermedia and web applications - SUE and MiLE - developed by the authors' research teams. It also borrows some concepts from various "general" usability evaluation methods (heuristic evaluation, scenario driven evaluation, cognitive walkthrough, task based testing). The main purpose of MiLE+ is to be more systematic and structured than its "inspirators", and to be particularly suited for novice evaluators.
19. Multiple-User Simultaneous Testing (MUST)	(Paul, Yadamsuren, & Erdelez, 2012)	Multiple-User Simultaneous Testing (MUST) is a technique that involves data collection from a group of users simultaneously. The opportunity for MUST may emerge when the real users of the system gather together for an event and they can be approached to participate.

20. The new hybrid model (NHM)	(Dominic, Jati, & Hanim, 2013)<br>(Rekik & Kallel, 2011)	The new hybrid model (NHM) has been implemented using combination of LWM (linear weightage model) and FAHP (Fuzzy Analytical Hierarchy Process) to generate the weights for the criteria which are better and more fairly preference.
21. PowerMapper	(Cherfi, Tuan, & Comyn-Wattiau, 2014)	PowerMapper provides a collection of tools to detect errors concerning broken links, browser compatibility, accessibility etc. It is based on WCAG standards (Web Content Accessibility, W3C organization).
22. Programmsystem zur kommunikationsergonomischen Untersuchung rechnerunterstützter Verfahren (PROKUS)	(Alva et al., 2008)	PROKUS (Programmsystem zur kommunikationsergonomischen Untersuchung rechnerunterstützter Verfahren) is a computer-system designed to carry out of usability evaluations according to different evaluation situations.
23. Preference Ranking Organization Method for Enrichment Evaluations (PROMETHEE)	(Kaya, 2010)<br>(Tsai, Chou, & Lai, 2010)	PROMETHEE (Preference Ranking Organization Method for Enrichment Evaluations) is an outranking method for a finite set of alternative actions to be ranked and selected among criteria, which are often conflicting. PROMETHEE is also a quite simple ranking method in conception and application compared with the other methods for multi-criteria analysis.
24. Quality Evaluation Method (QEM)	(Cherfi, Tuan, & Comyn-Wattiau, 2014)<br>(Rekik & Kallel, 2011)	Website Quality Evaluation Method (QEM) is a tool that has been customised to assess the quality of academic websites. Particularly, the purpose was to obtain a ranking for numerous academic sites. The ISO 9126 standard characteristics were used to evaluate the quality.
25. QualWeb Evaluator	(Cherfi, Tuan, & Comyn-Wattiau, 2014)	QualWeb evaluator consists of a tool for testing web pages/applications, before and after the web browsing processing, and displaying the results of the evaluation, according to both types of processing.
26. Questionnaire for User Interaction Satisfaction (QUIS)	(Alva et al., 2008)	The Questionnaire for User Interaction Satisfaction (QUIS) is a measurement tool designed to assess a computer user's subjective satisfaction with the human-computer interface.
27. Suitability Assessment of Materials (SAM)	(Janiak, Rhodes, & Foster, 2013)	Suitability Assessment of Materials (SAM) is an instrument that rates materials on 22 factors to evaluate the following: (1) content, (2) literacy demand, (3) graphics, (4) layout and typography, (5) learning stimulation and motivation, and (6) cultural appropriateness.

28.SERVQUAL	(Swaid & Wigand, 2007)	The SERVQUAL model has five dimensions: tangibles (appearance of physical facilities, equipment, personnel and communication materials), reliability (ability to perform the promised service dependably and accurately), responsiveness (willingness to help customers and provide prompt services), assurance (knowledge and courtesy of employees and their ability to convey trust and confidence) and empathy (the caring and individualized attention provided to the customers) (Parasuraman et al. 1988). The SERVQUAL instrument has high acceptance and reliability across the spectrum of different industries such as traditional stores, healthcare, tourism, festivals, the automobile industry and information systems.
29.Software Usability Measurement Inventory (SUMI)	(Carlos Flavián, Miguel Guinalíu, & Raquel Gurrea, 2006)	The Software Usability Measurement Inventory (SUMI) is a rigorously tested and proven method of measuring software quality from the end user's point of view. It involves a questionnaire which has been developed, validated, and standardised in a wide selection of languages.
30.System Usability Scale (SUS)	(Carlos Flavián, Miguel Guinalíu, & Raquel Gurrea, 2006)	The System Usability Scale (SUS) provides a “quick and dirty”, reliable tool for measuring the usability. It consists of a 10 item questionnaire with five response options for respondents; from Strongly agree to Strongly disagree. It allows you to evaluate a wide variety of products and services, including hardware, software, mobile devices, websites and applications.
31.Task-Technology Fit Model (TTF)	(Wang, Wang, & Wei, 2014)	Based on the task-technology fit theory (TTF) and the technology-to-performance chain, the task-technology fit model addresses the importance of the fit among technology, task, and individual characteristics in terms of determining the performance of technology use.
32.TAW	(Cherfi, Tuan, & Comyn-Wattiau, 2014)	TAW is a tool that detects a list of problems classified according to four categories: Perceivable, Operable, Unders- tandable and Robust.
33.Technique for Order Preference by Similarity to an Ideal Solution (TOPSIS)	(Kaya, 2010)<br>(Tsai, Chou, & Lai, 2010)	Technique for Order Preference by Similarity to an Ideal Solution (TOPSIS) is based on an aggregating function representing “closeness to the ideal” and is used to eliminate the units of criterion functions. The TOPSIS method determines a solution with the shortest distance to the ideal solution and the greatest distance from the negative-ideal solution, but it does not consider the relative importance of these distances.

34. VlseKriterijumska Optimizacija I Kom- promisno Resenje (VIKOR)	(Chen, Tzeng, & Chang, 2015)<br>(Kaya, 2010)<br>(Tsai, Chou, & Lai, 2010)	VlseKriterijumska Optimizacija I Kom- promisno Resenje (VIKOR) is a method used to evaluate and rank websites. This method utlisies an aggregating function, which then represents the site's distance from an ideal solution. This ranking index is an aggregation of all criteria, including the relative importance of criteria and a balance between total and individual satisfaction.
35. Website Analysis and Measurement Inventory (WAMMI)	(Alva et al., 2008)	Website Analysis and Measurement Inventory (WAMMI) is used to assess: website user experience; benchmark the website against existing databases; tracks changes to website user experience over time, researches visitors to the site and what they think.
36. Web Application Quality Evaluation (WAQE)	(Cherfi, Tuan, & Comyn-Wattiau, 2014)	Web Application Quality Evaluation (WAQE) model is based on two axons: internal (within the organisation) and external (the users). The model places emphasis on quality issues as defined by ISO 9126 and other web quality factors and utilises importance-based criteria for evaluating requirements.
37. WAVE	(Cherfi, Tuan, & Comyn-Wattiau, 2014)	WAVE provides the user with reports using icons, structures and texts to help find errors in a website. It is an automated, freely available web accessibility evaluation tool, provided by WebAIM.
38. Web Q-Model	(Cherfi, Tuan, & Comyn-Wattiau, 2014)	Web Q-Model is a general and holistic model, easy to apply and scalable for different domains, aimed at helping web designers to develop accurate and quick websites evaluation.
39. WebQEM	(M. Arrue, Fajardo, López, & Vigo, 2007)<br>(Cherfi, Tuan, & Comyn-Wattiau, 2014)	WebQEM is a quantitative evaluation strategy to assess website and application quality, as defined in the ISO/IEC 9126–1 standard.
40. WebQual	(Cherfi, Tuan, & Comyn-Wattiau, 2014)<br>(Longstreet, 2010)	WebQual, a website quality measure with 12 dimensions, used to assesses the usability, information, and service interaction quality of websites.
41. WebTango	(Dominic, Jati, & Hanim, 2013)	The WebTango is a quality checker tool, which proposes to help non-professional designers to develop their sites using quantitative measures of the navigational, informational and graphical aspects of a website. The usability evaluation approach is used in the field of the software engineering and adapted to the website usability evaluation.
42. Website Evaluation Questionnaire (WEQ)	(Elling, Lentz, de Jong, & van den Bergh, 2012)	The Website Evaluation Questionnaire (WEQ) is a valid and reliable instrument with seven clearly distinct dimensions.

43.Web Quality Model (WQM)	(Cherfi, Tuan, & Comyn-Wattiau, 2014)	Web Quality Model (WQM) structures the characteristics according to three dimensions: features, quality characteristics, and life cycle processes.
